# Sense of coherence, mental well-being and perceived preoperative hospital and surgery related stress in surgical patients with malignant, benign, and no neoplasms

**DOI:** 10.1186/s12888-020-02953-x

**Published:** 2020-11-27

**Authors:** Henning Krampe, Ute Goerling, Claudia D. Spies, Sina K. Gerhards, Sören Enge, Anna-Lena Salz, Léonie F. Kerper, Tatjana Schnell

**Affiliations:** 1 Department of Anesthesiology and Operative Intensive Care Medicine (CCM, CVK), Charité - Universitätsmedizin, corporate member of Freie Universität Berlin, Humboldt-Universität zu Berlin, and Berlin Institute of Health, Charitéplatz 1, 10117 Berlin, Germany; 2 Charité Comprehensive Cancer Center, Charité - Universitätsmedizin, corporate member of Freie Universität Berlin, Humboldt-Universität zu Berlin, and Berlin Institute of Health, Berlin, Germany; 3 Department of Psychology, Faculty of Natural Sciences, Medical School Berlin, Berlin, Germany; 4 Department of Anaesthesiology, Intensive Care, Emergency and Pain Medicine, Hospital Wolfenbuettel gGmbH, Wolfenbuettel, Germany; 5 Institute of Psychology, University of Innsbruck, Innsbruck, Austria; 6 Norwegian School of Theology, Religion and Society, Oslo, Norway

**Keywords:** Cancer, Depression, Mediation analysis, Mental well-being, Neoplasm, Preoperative, Sense of coherence, Stress

## Abstract

**Background:**

This prospective, cross-sectional, observational study examined associations between sense of coherence (SOC), mental well-being, and perceived preoperative hospital and surgery related stress of surgical patients with malignant, benign, and no neoplasms. The objective was to assess a putative association between SOC and preoperative stress, and to test for a statistical mediation by mental well-being.

**Method:**

The sample consisted of 4918 patients from diverse surgical fields, of which 945 had malignant neoplasms, 333 benign neoplasms, and 3640 no neoplasms. For each subsample, we conducted simple mediation analyses to test an indirect effect of SOC on preoperative stress mediated by mental well-being. The models were adjusted for age, gender, and essential medical factors.

**Results:**

Patient groups did not differ significantly regarding degrees of SOC and mental well-being (SOC, M [SD]: 12.31 [2.59], 12.02 [2.62], 12.18 [2.57]; mental well-being M [SD]: 59.26 [24.05], 56.89 [22.67], 57.31 [22.87], in patients with malignant, benign, and without neoplasms, respectively). Patients without neoplasms reported significantly lower stress (4.19 [2.86], M [SD]) than those with benign (5.02 [3.03], M [SD]) and malignant neoplasms (4.99 [2.93], M [SD]). In all three mediation models, SOC had significant direct effects on stress, with higher SOC being associated with lower stress (− 0.3170 [0.0407], − 0.3484 [0.0752], − 0.2919 [0.0206]; c’ [SE], *p* < 0.001 in patients with malignant, benign, and without neoplasms, respectively). In patients with malignant neoplasms and without neoplasms, SOC showed small indirect effects on stress that were statistically mediated by well-being. Higher SOC was related to higher well-being, which in turn was related to lower stress. In patients with benign neoplasms, however, no significant indirect effects of SOC were found.

**Conclusions:**

SOC was directly associated with lower perceived hospital and surgery related stress, over and above the direct and mediation effects of mental well-being. Because the data are cross-sectional, conclusions implying causality cannot be drawn. Nevertheless, they indicate important relationships that can inform treatment approaches to reduce elevated preoperative stress by specifically addressing low SOC.

**Trial registration:**

clinicaltrials.gov Identifier: NCT01357694. Registered 18 May 2011

## Background

Perceived preoperative stress is common among surgical patients, and it has mainly been investigated in the form of acute preoperative anxiety [1–6]. In contrast to general emotional distress, the experience of acute preoperative stress is particularly related to unpleasant feelings concerning the impending surgery and hospital treatment [2, 4, 7–9]. Preoperative stress can by itself be regarded as a negative patient reported outcome [2, 4]. Moreover, it is associated with peri- and postoperative complications and worse surgical outcomes [2, 7, 9–16]. So far, oncological studies have rarely investigated preoperative stress as primary outcome. Instead, numerous studies have focused on general emotional distress unrelated to surgery, which has been a major target of both clinical practice and psycho-oncological research for a long time [17–21]. Little is known about the relative degree of distress experienced by cancer patients before surgery compared to non-cancer patients. Three studies found higher state anxiety in cancer patients [22–24], while another study did not find any significant differences [25].

Previous studies demonstrated that several patient characteristics and clinical factors were associated with preoperative stress, such as gender [22, 25, 26], mental health [22], preoperative physical health [22], surgical field [1] and extent of surgical procedure [5, 27]. Further knowledge on related factors may contribute to both our understanding of preoperative stress and the improvement of treatment approaches. Individual resources like sense of coherence (SOC) that may help patients to cope with stress are of particular interest. SOC is defined as a person’s view of the world in terms of a basic confidence that one’s own life is comprehensible, manageable, and meaningful [28, 29]. Positive associations of SOC with health and quality of life have frequently been shown [30–32]. According to the salutogenetic model by Antonovsky, people with a high SOC are more successful in coping with stressful life events and problems, which results in less perceived distress and better health. SOC has shown robust associations with aspects of mental health like quality of life, mental well-being, and clinical symptoms of anxiety and depression [29–31, 33]. A recent meta-analysis found that in cancer patients, there is a substantial negative association of SOC and general emotional distress, which is independent of age, gender, disease stage, and days since diagnosis [33]. To our knowledge, there are no studies on the relationship of SOC and acute perceived preoperative stress in surgical patients with cancer, but it seems likely that SOC and preoperative stress are also negatively related.

Concerning the role of mental well-being, it might be hypothesized that SOC results in higher mental well-being and this in turn reduces distress like, e.g., acute preoperative stress. In this case, mental well-being would serve as a mediator between SOC and low acute preoperative stress. Because the present investigation is based on cross-sectional data, causal relationships cannot be established [34, 35]. Two lines of reasoning would support the mentioned direction, however. One is the empirically validated assumption that SOC and meaning in life, which Antonovsky claimed to be the most important element in SOC [28], actually *affect* subjective well-being and stress perception [36–39], as well as functional health outcomes [40–42]. Additionally, one might bring forward the argument that the most stable factor would best represent the independent variable. Both SOC and meaningfulness are psychological dispositions that have a tendency to increase over the lifespan, but they are known to be relatively stable over several years [39, 43–46]. The extent of mental well-being is, similar to symptoms of anxiety and depression, less stable and changes over medium-term periods of time [47–49], while acute preoperative stress is a state variable that changes within short-term intervals [9, 50]. It is therefore plausible to conceive of SOC as the independent factor, acute perceived preoperative stress as the dependent variable, and mental well-being as a potential mediating factor.

This study examined associations between SOC, mental well-being, and perceived preoperative hospital and surgery related stress of surgical patients with malignant, benign, and no neoplasms. The objective was to assess a putative association between SOC and preoperative stress, and to test for a statistical mediation by mental well-being.

## Methods

### Setting, study design, ethics approval and consent to participate

This cross-sectional, prospective, observational study conducted secondary mediator analyses on data from the research project Bridging Intervention in Anesthesiology (BRIA).

We investigated three groups of surgical patients, those with malignant, benign, and no neoplasms. For each patient group, we conducted a simple mediation model that tested the indirect effect of SOC on perceived hospital and surgery related stress mediated through mental well-being. The models were adjusted for demographic and clinical factors that are potentially related to perceived preoperative stress. These covariates included age, gender, preoperative physical health, surgical field, severity of medical comorbidity, as well as extent of surgical procedure. In the samples of patients with neoplasms, additional analyses were conducted including tumor site as covariate. In order not to confuse preoperative stress with preoperative symptoms of clinical anxiety, we explicitly assessed perceived stress with items relating to the impending surgery and treatment in hospital.

The BRIA project was approved by the Institutional Review Board of Charité – Universitäts medizin Berlin [EA1/014/11], registered with ClinicalTrials.gov [Identifier: NCT01357694], and conducted in compliance with the Helsinki Declaration. All participants provided written informed consent for all procedures. The full details of the setting, patient recruitment and assessment instruments are available in our previous publications [51, 52].

### Data collection and eligibility criteria

By means of a computer-assisted psychosocial self-assessment, we collected patient-reported data in the preoperative assessment clinics of the Charité – Universitätsmedizin Berlin, Campus Charité Mitte and Campus Virchow Klinikum, Berlin, Germany, from May 2011 to August 2012.

Six months after preoperative assessment, we obtained medical data from the electronic patient data management system of the hospital, and determined six measures to assess essential medical parameters. All categories of these medical measures are shown in Table 1; more details are available in our previous publications [53, 54]. The six parameters included:

**Table 1:** Demographic and clinical characteristics of patient sample (*N* = 4918) and comparison of patients with malignant neoplasms (A), benign neoplasms (B), and without neoplasms (C); n (%), mean [SD]

	A. Malignant neoplasms *n* = 945	B. Benign neoplasms *n* = 333	C. Without neoplasms *n* = 3640	***p***
Age (Years)	55.49 [14.08]	44.53 [12.30]	44.57 [15.64]	A-B-C < 0.001; A-B < 0.001; A-C < 0.001; B-C = 0.952
Men	553 (58.50)	86 (25.80)	1905 (52.30)	A-B-C < 0.001; A-B < 0.001; A-C = 0.001; B-C < 0.001
Sense of Coherence (BASOC) ^b^	12.31 [2.59]	12.02 [2.62]	12.18 [2.57]	A-B-C = 0.176; A-B = 0.084; A-C = 0.170; B-C = 0.283
Mental wellbeing (WHO-5) ^a^	59.26 [24.05]	56.89 [22.67]	57.31 [22.87]	A-B-C = 0.058; A-B = 0.117; A-C = 0.026; B-C = 0.746
Clinically significant depression ^a^	307 (32.50)	120 (36.00)	1266 (34.80)	A-B-C = 0.340; A-B = 0.238; A-C = 0.186; B-C = 0.645
Perceived hospital and surgery related stress ^c^	4.99 [2.93]	5.02 [3.03]	4.19 [2.86]	A-B-C < 0.001; A-B = 0.873; A-C < 0.001; B-C < 0.001
Elevated perceived hospital and surgery related stress ^c^	517 (54.7)	183 (55.0)	1529 (42.0)	A-B-C < 0.001; A-B = 0.938; A-C < 0.001; B-C < 0.001
Length of hospital stay (days)	6.99 [8.63]	3.91 [3.65]	4.10 [5.77]	A-B-C < 0.001; A-B < 0.001; A-C < 0.001; B-C = 0.559
Physical health (ASA Classification) ^d^				
ASA I, II	733 (77.60)	302 (90.7)	3206 (88.10)	A-B-C < 0.001; A-B < 0.001; A-C < 0.001; B-C = 0.156
ASA III, IV	212 (22.40)	31 (9.3)	434 (11.90)	
Surgical field				
Neuro-, head and neck surgery	126 (13.30)	92 (27.60)	1155 (31.70)	A-B-C < 0.001; A-B < 0.001; A-C < 0.001; B-C < 0.001
Abdomino-thoracic surgery	726 (76.80)	194 (58.30)	935 (25.70)	
Peripheral surgery	93 (9.80)	47 (14.10)	1550 (42.60)	
Site of tumor				
Head and neck	85 (9.00)	61 (18.30)		A-B < 0.001
Breast	87 (9.20)	9 (2.70)		
Female genital organs	171 (18.10)	152 (45.80)		
Skin	80 (8.50)	32 (9.60)		
Lung	7 (0.70)	0 (0)	–	
Prostate	234 (24.80)	0 (0)		
Urinary system	97 (10.30)	13 (3.90)		
Bowel	28 (3.00)	1 (0.30)		
Nervous system	24 (2.50)	18 (5.40)		
Pancreas	12 (1.30)	1 (0.30)		
Hepatobiliary system	22 (2.30)	2 (0.60)		
Stomach / esophagus	18 (1.90)	1 (0.30)		
Lymphatic system	14 (1.50)	0 (0)		
Other	66 (7.00)	43 (12.90)		
Medical comorbidity (CCI) ^e^				
0 ‘None’	93 (9.8)	277 (83.2)	2952 (81.1)	A-B-C < 0.001; A-B < 0.001; A-C < 0.001; B-C = 0.763
1 ‘Low’	384 (40.60)	44 (13.20)	548 (15.10)	
2 ‘Moderate’	216 (22.90)	8 (2.40)	103 (2.80)	
3 ‘High’	252 (26.70)	4 (1.20)	37 (1.00)	
Extent of surgical procedure (POSSUM operative severity) ^f^				
1 ‘Minor’	256 (27.10)	131 (39.3)	1467 (40.30)	A-B-C < 0.001; A-B < 0.001; A-C < 0.001; B-C = 0.001
2 ‘Moderate’	413 (43.70)	86 (25.80)	1112 (30.50)	
4 ‘Major’	145 (15.30)	106 (31.80)	845 (23.20)	
8 ‘Major +’	131 (13.90)	10 (3.00)	216 (5.90)	

^a^WHO-5 (World Health Organization 5-item Well-Being Index) with lower scores indicating lower wellbeing (range: 0–100); clinically significant depression according to WHO-5 sum score cut-off ≤50

^b^BASOC (Brief Assessment of Sense of Coherence) with higher scores indicating higher sense of coherence (range 3–15)

^c^Perceived hospital and surgery related stress distress with higher scores indicating higher stress (range 0–10); scores ≥5 indicate elevated stress

^d^ASA classification (American Society of Anesthesiologists). ASA I, II: Healthy patients (ASA I), and patients with mild systemic disease, no functional limitations (ASA II); ASA III, IV: Patients with severe systemic disease with definite functional limitation (ASA III) and patients with severe systemic disease that is a constant threat to life (ASA IV)

^e^CCI (Charlson Comorbidity Index)

^f^POSSUM (Physiological and Operative Severity Score for the enUmeration of Mortality and Morbidity)

1. Overall indicator of physical health status: Evaluation of patients’ perioperative risk according to the ASA (American Society of Anesthesiologists) physical status classification system [55, 56].

2. Surgical field: Neuro-, head, and neck surgery; abdomino-thoracic surgery; peripheral surgery [53, 57].

3. Severity of medical comorbidity: Charlson Comorbidity Index (CCI) [58].

4. Extent of surgical procedures: Item ‘operative severity’ of the POSSUM scoring system (Physiological and Operative Severity Score for the enUmeration of Mortality and Morbidity) [59, 60].

5. Site of tumor: Based on ICD-10 codes of patients’ primary diagnoses, 14 sites of tumor were determined for both malignant and benign tumors.

6. Status of tumor: Malignant, benign, and suspect neoplasms.

**Fig. 1:**
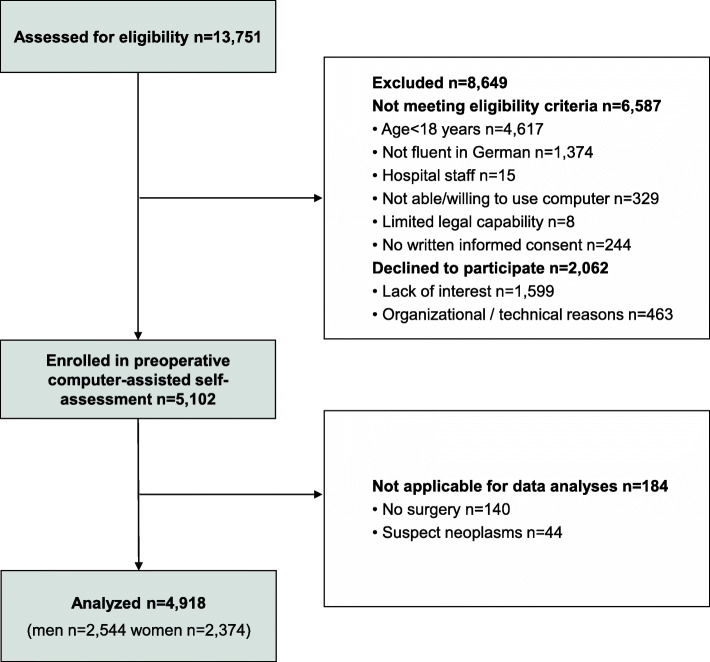
Flow diagram of study participants

Inclusion criteria were written informed consent to participate after having been properly instructed; patient of the preoperative anesthesiological assessment clinic, and age above 17 years. Exclusion criteria were surgery with an emergency or urgent indication; inability to attend the preoperative assessment clinic; insufficient knowledge of German language; being a member of the hospital staff; admitted in police custody; accommodation in an institution by official or court order; being under guardianship, and psychiatric, neurological or other conditions associated with limited legal capability or limited capability of being properly instructed or giving informed consent. Figure 1 shows the flowchart of the inclusion process. Of the 5102 patients enrolled in the preoperative computer-assisted self assessment, data of 184 patients were not applicable for data analyses, either because planned surgeries were cancelled, or because of surgeries due to suspect neoplasms. In total, data of 4918 patients were included in the statistical analyses.

### Patient-reported outcome measures

Outcomes included measures of SOC, mental well-being, and perceived hospital and surgery related stress.

### Sense of coherence

We assessed SOC with the Brief Assessment of Sense of Coherence (BASOC) [61], a 3-item short version of the Sense of Coherence Scale 29 (SOC-29 [29]. The items are rated on a 5-point scale from 1 to 5, with sum scores ranging from 3 to 15, and higher scores indicating higher SOC. The BASOC has proven superior to the commonly used 3-item measure SOC-3 concerning reliability and validity [61]. With a Cronbach’s alpha of .71, the BASOC showed a comparatively good reliability for a short 3-item questionnaire measure; indicators of construct validity were also adequate in terms of a correlation of *r =* 0.77 between BASOC and SOC-29, excluding the three BASOC items, and correlations with different mental-health-related parameters between *r =* .45 to *r =* .63 [61]. In the present study, the internal consistency of the BASOC was even better than in the validation study by Schumann et al. (2003), with a Cronbach’s alpha of 0.74.

### Mental well-being

The short self-report questionnaire World Health Organization Well-Being Index (WHO-5) assesses subjective mental well-being over the past 2 weeks [47, 49, 62]. It consists of five items covering sensations of cheerfulness, calm, activity, freshness, and interest. Respondents rate how often they have experienced the described sensations on a 6-point scale from 0 (at no time) to 5 (all of the time), resulting in a raw sum score from 0 to 25, with higher scores indicating better well-being. The sum score is multiplied by 4 in order to yield a transformed scale score ranging from 0 to 100. The WHO-5 is also applied as a screening tool to identify depression by measuring decreased well-being. A sum score of 50 or less indicates clinically relevant depression [47, 49]. The WHO-5 has shown good internal consistency with Cronbach’s alphas between .84 and .95 [47, 63] and good retest reliability, as indicated by an intraclass correlation coefficient of .81 [48]. A recent systematic literature review found numerous articles demonstrating that the WHO-5 has high validity as both a measure of mental well-being as well as a screening tool for clinically significant depression [49]. In the present study, the internal consistency of the WHO-5 is high, with a Cronbach’s alpha of .87.

### Perceived preoperative hospital and surgery related stress

The perceived preoperative hospital and surgery related stress was measured with an adapted version of the Distress Thermometer [64], using two vertical numerical rating scales from 0 (no stress at all) to 10 (extreme stress) illustrated as a thermometer. The first item had the instruction “Please choose the number (0 to 10) that best represents the stress you are experiencing because of the upcoming hospital stay”, and the second item had the instruction “Please choose the number (0 to 10) that best represents the stress you are experiencing because of the upcoming surgery”. The two items correlated highly with each other with a Pearson correlation coefficient of *r =* 0.87. Thus, they were added up and their mean sore was used in the analysis. Exploratory analyses in a subset of the sample (*n* = 2320) showed high correlations of state anxiety, measured by the state scale of the State Trait Anxiety Inventory (STAI) [65], with the hospital related distress item (*r =* 0.59), the surgery related distress item (*r =* 0.62), and the mean score of the two items (*r =* 0.63). For an exploratory estimate of elevated perceived preoperative stress we used a cut-off point of ≥5, as has been recommended for visual analogue scales of preoperative anxiety [66].

### Statistical analyses

Our primary objective was to investigate to what extent the putative relation between SOC and perceived preoperative hospital and surgery related stress would be mediated by mental well-being in surgical patients with malignant neoplasms, benign neoplasms, and no neoplasms. We conducted three simple mediation analyses using the PROCESS macro, version 3.2, for SPSS [34, 35]. The analyses were based on two multiple linear regression models. Both models simultaneously included the independent factor SOC, as measured with the BASOC sum score, and the covariates age in years, gender, preoperative physical health (ASA), surgical field, severity of medical comorbidity (CCI), as well as extent of surgical procedure (POSSUM operative severity item). In the two samples of patients with neoplasms, analyses were first conducted without adjusting for tumor site, and thereafter with tumor site as covariate. Categorical covariates with more than two categories were dummy coded before being entered as several dummy variables in the regression models. In order to avoid calculation errors in bootstrapping analyses, tumor site categories with fewer than 8 cases were collapsed into the category ‘other’.

**Fig. 2:**
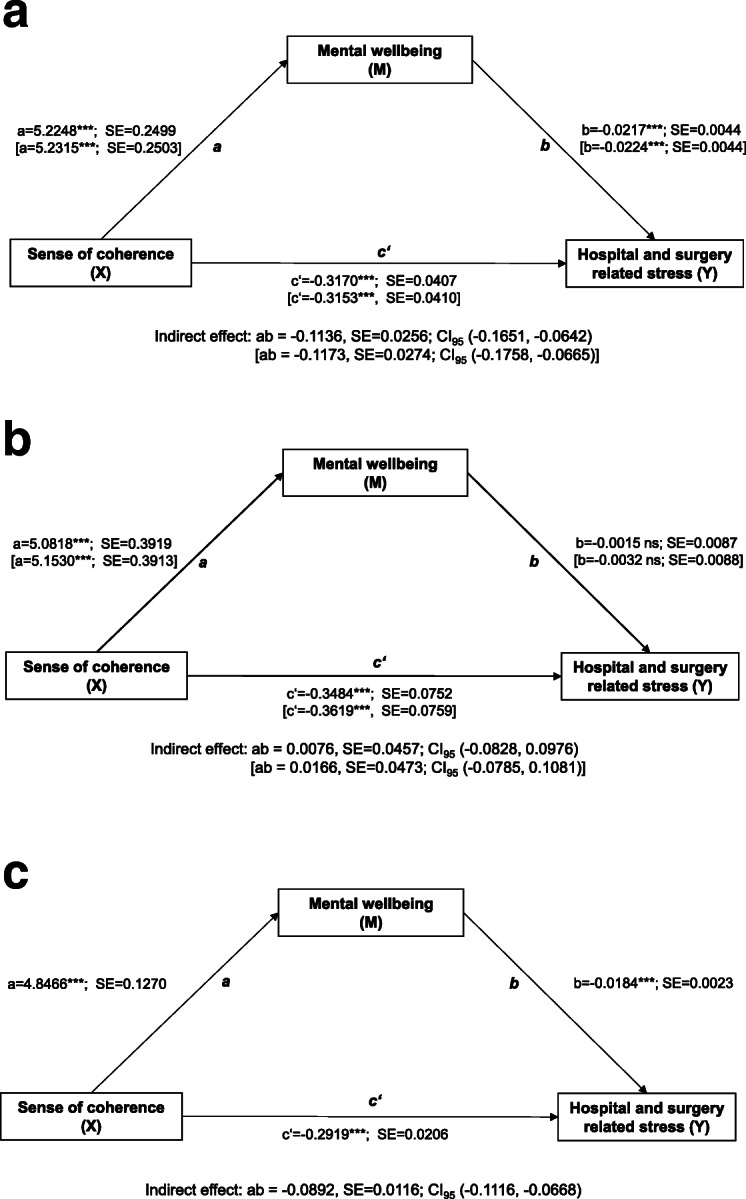
**a**, **b**, **c**. Mediation models of the effect of sense of coherence (SOC) (X) on perceived hospital and surgery related stress (Y) through mental well-being (M). Regression models adjusted for age, gender, physical health (ASA classification), surgical field, medical comorbidity (CCI), and extent of surgical procedure (POSSUM operative severity item). Data in brackets: Regression model additionally adjusted for site of tumor. **a**. Patients with malignant neoplasms (*n =* 945). **b**. Patients with benign neoplasms (*n =* 333). **c**. Patients without neoplasms (*n* = 3640)

The first regression model predicted mental well-being as measured with the WHO-5 sum score. The second model included the WHO-5 sum score in the set of predictor variables and predicted perceived preoperative hospital and surgery related stress. The different relationships of the factors of the mediation analyses are shown by path diagrams illustrating the unstandardized ordinary least squares (OLS) regression coefficients of the regression models (Fig. 2 a, b and c). Paths *a* and *c’* demonstrate the direct effects of the independent variable X, SOC, on the mediator, mental well-being, and on the dependent variable Y, perceived preoperative hospital and surgery related stress, respectively. Path *b* refers to the direct effect of the mediator, mental well-being, on the dependent variable. This effect is a partial regression coefficient that is controlling for the independent variable, SOC. The indirect effect of SOC on perceived preoperative hospital and surgery related stress, mediated by mental well-being, is determined by the product of the paths a*b. Indirect effects were tested with a percentile bootstrapping approach based on 5000 bootstrap samples [34]. An indirect effect is considered statistically significantly different from zero when the 95% bootstrap confidence intervals of the product a*b do not include 0. As effect sizes of the indirect and direct effects of SOC, we calculated the completely standardized effects ab_cs_ and c’_cs_ with 95% bootstrap confidence intervals. The completely standardized effect expresses the indirect and direct effects of the independent variable X, here SOC, as differences in standard deviations (SD) of the dependent variable Y, here hospital and surgery related distress. The effect sizes are defined as ab_cs_ = SD_x_*(ab_ps_) and c’_cs_ = SD_x_*(c’_ps_), where ab_ps_ = a*b/SD_y_ and c’_ps_ = c’/SD_y_ [34].

Descriptive results were expressed as relative frequencies in percent, as well as means (M) and standard deviations (SD). Bivariate correlations were tested using Pearson correlation analyses. The comparisons of patient groups with malignant neoplasms, benign neoplasms, and no neoplasms regarding demographic and clinical characteristics were conducted with Chi-square tests for categorical variables, as well as with one-way ANOVAs for all three groups, and independent-samples T-tests for pairs of groups, for metric variables. A two-tailed *p*-value < 0.05 was considered statistically significant for all statistical tests except the bootstrapping method.

## Results

Out of the 4918 study participants, 945 underwent surgery due to malignant neoplasms, 333 due to benign neoplasms, and 3640 had surgeries due to other diseases. Demographic, psychological, and clinical characteristics of the three patient groups are presented in Table 1. Patients with malignant neoplasms were older and had a higher rate of men than those with benign and no neoplasms. The groups did not differ significantly regarding either SOC or mental well-being. However, patients without neoplasms had significantly lower mean perceived hospital and surgery related stress, as well as lower rates of elevated stress than those with neoplasms. Concerning preoperative physical health, medical comorbidity, length of hospital stay, and extent of surgical procedure, the data indicated that the group with malignant neoplasms had significantly higher rates of severely impaired patients. The three groups differed significantly regarding the distribution of the surgical fields neuro-, head, and neck surgery, abdomino-thoracic surgery, as well as peripheral surgery, reflecting the diversity of included surgical patients. Finally, patients with malignant and benign neoplasms showed different distributions of sites of tumors.

In all three patient groups, SOC and mental well-being showed large positive bivariate correlations (.54 to .61) (Table 2). While perceived hospital and surgery related stress was moderately negatively related to SOC in all groups (−.34 to −.42), its correlations with mental well-being varied between −.23 in patients with benign neoplasms and − 0.38 in those with malignant neoplasms (Table 2).

**Table 2:** Correlations between sense of coherence, well-being, and perceived hospital and surgery related distress (*N* = 4918)

	SOC	Mental well-being
**A.** Patients with malignant neoplasms (*n =* 945)		
Mental well-being	0.59^***^	
Perceived hospital and surgery related stress	−0.42^***^	− 0.38^***^
**B.** Patients with benign neoplasms (*n =* 333)		
Mental well-being	0.61^***^	
Perceived hospital and surgery related stress	−0.34^***^	−0.23^***^
**C.** Patients without neoplasms (*n =* 3640)		
Mental well-being	0.54^***^	
Perceived hospital and surgery related stress	−0.37^***^	−0.31^***^

*** *p* < 0.001

### Simple mediation analyses

Figure 2a, b, and c show the results of three simple mediation models testing indirect effects of SOC on perceived hospital and surgery related stress mediated by mental well-being. In all three models, SOC showed significant direct effects on stress, with higher SOC being associated with lower stress. The corresponding direct effect sizes c’_cs_ were − 0.2808 [− 0.2793], − 0.3014 [− 0.313], and − 0.2616 for the samples of patients with malignant, benign, and no neoplasms, respectively; effect sizes in brackets refer to the models additionally adjusted for sites of tumor. In patients with malignant neoplasms and without neoplasms, SOC showed small significant indirect effects on stress that were statistically mediated by mental well-being. Higher SOC was related to higher well-being which was, in turn, related to lower stress. The corresponding indirect effect sizes ab’_cs_ were − 0.1006, CI_95_ (− 0.1456, − 0.0568), [− 0.1039, CI_95_ (− 0.1543, − 0.0592)], and − 0.0800, CI_95_ (− 0.1004, − 0.0596), for patients with malignant and no neoplasms, respectively. In patients with benign neoplasms, no significant indirect effects of SOC were found; accordingly, effect sizes ab’_cs_ were small: 0.0065, CI_95_ (− 0.0723, 0.0844), [0.0143, CI_95_ (− 0.0684, 0.0930)].

## Discussion

This study investigated direct and indirect effects of SOC on perceived preoperative hospital and surgery related stress. Three simple mediation models with mental well-being as putative mediator were conducted in patients who underwent surgery due to malignant or benign neoplasms, or due to other diseases. In all three models, SOC showed substantial direct effects on perceived stress. Partly, SOC also showed indirect effects, although those were much smaller. As expected, patients who had a high SOC were less likely to experience preoperative hospital and surgery related stress. They also reported better mental well-being. In patients with benign neoplasms, however, this did not, and in those with malignant or no neoplasms, it did only slightly account for the relationship between SOC and lower preoperative stress.

### Preoperative stress in surgical patients with cancer

To our knowledge, there are only few investigations comparing preoperative hospital and surgery related stress in patients with and without cancer. In the present study, patients with malignant, benign, and no neoplasms did not differ significantly regarding degrees of SOC, mental well-being, or clinically relevant depressive symptoms. However, patients who underwent surgery due to diseases without neoplasms reported significantly lower mean stress scores and lower rates of elevated hospital and surgery related stress than those who underwent surgery due to malignant and benign neoplasms. These results suggest that suffering from a neoplasm may be associated with clinically relevant and detrimental acute stress, but not necessarily with distress inducing lower SOC and / or lower mental-well-being. Our data are inconsistent with the findings of Domar et al. (1998) [25], but comparable with those of three previous studies that found higher preoperative state anxiety in cancer patients compared to non-cancer patients [22–24].

Research on preoperative stress is scarce in cancer patients. Preoperative stress was rarely assessed with explicit reference to the surgical and/or hospital context e.g. [67]. However, several studies measured unspecified state anxiety e.g. [22–24, 68–70], or general emotional distress before surgery e.g. [24, 71–75]. Altogether, preoperative stress in cancer patients was found to be related to adverse peri- and postoperative outcomes like higher pain, worse health-related quality of life, and increased incidence of delirium. These results resemble those of studies that did not explicitly focus on surgical patients with cancer [2, 7, 9–16]. While our investigation did not examine associations between stress and postoperative outcomes, it adds to the research on potential predictors of stress in cancer patients. The results suggest that the stress-reducing effect of SOC is not substantially mediated by an improvement of mental well-being, and that there might be other relevant factors to explain how SOC may relieve preoperative stress.

### Meaning in life as a specific resource of the stress buffering effect of SOC

Our findings support the assumption that an understanding of one’s life as meaningful, comprehensible, and manageable is directly linked to efficient stress regulation. This might be due to a stress buffering effect, allowing patients to draw on available resources to deal with the challenging situation of an upcoming surgery. Importantly, the direct effects of SOC on preoperative stress are of relatively similar seize in surgical patients with malignant, benign, and no neoplasms, supporting Antonovsky’s conception that the associations of SOC and health-related factors are comparable over diverse diseases [28, 29]. Thus, the question arises which specific psychological processes might be associated with the stress buffering effect of SOC. A large body of research has been carried out on effects of the central factor of SOC, meaning in life. These studies suggest that meaning in life activates a variety of psychological processes that are helpful for a successful coping with stressful life events and problems, such as better self-regulation skills, higher hopefulness and optimism, as well as experiences of competence, self-determination, and social integration [76–79].

### Limitations and future directions

This study applied the BASOC to assess SOC. Like with other SOC measures, results based on the BASOC cannot be clearly attributed to one or more of the theorized underlying SOC facets of meaningfulness, comprehensibility, and manageability. Further research should use more specific measures, e.g. the meaningfulness and crisis of meaning scales from the Sources of Meaning and Meaning in Life Questionnaire, SoMe [45, 80]. Another limitation is the correlational design of the study that precludes conclusions concerning causality. SOC, mental well-being and preoperative stress were assessed preoperatively at the same time. Hence, without testing the mediation hypothesis with longitudinal data, no conclusions can be drawn concerning the causality of the relationship between SOC, mental well-being and preoperative stress; we can only claim a statistical mediation at this point [81]. Furthermore, our mediation models did not comprise additional oncological parameters as covariates, e.g. disease stage and time since cancer diagnosis. However, the models were adjusted for age and gender, as well as for substantial medical and surgical factors including physical health, surgical field, medical comorbidity, extent of surgical procedure, and site of tumor. As already mentioned, previous research did not find any moderator effects of disease stage and time since cancer diagnosis on the relationship between SOC and meaning in life, respectively, and psychological distress [33]. In the study at hand, the direct effects of SOC on stress were comparable in patients with malignant, benign, and no neoplasms. Both findings may suggest that the psychological processes associated with the relations of SOC and psychological outcomes might rather be independent of types of diseases and disease stages.

Taken together, future studies that aim to conduct analyses of effects of personal resources on acute stress in cancer patients should employ more specific measures and establish temporal precedence of the factors of the mediation model. Additionally, it would be interesting to explore whether the findings are robust when additional oncological patient characteristics are included as covariates in mediation models. Regarding relevant factors to explain how and under which conditions meaning in life and SOC may relieve preoperative stress, further potential mediators and moderators should be investigated, including factors like social support, self-regulation, socioeconomic status, diverse health behaviors, and measures of subjective health.

## Conclusions

In this study, the rate of elevated perceived hospital and surgery related stress was relatively high, with 54.7, 55.0, and 42.0% in patients with malignant, benign, and no neoplasms, respectively. Contemporary approaches conceive of elevated preoperative stress as a clinically relevant negative patient reported outcome, irrespective of whether it is associated with worse postoperative surgical outcomes [2, 4]. This perspective is consistent with psycho-oncological approaches that, independently of an upcoming surgery, consider emotional distress as a vital sign that requires treatment to improve the patients’ mental health and health-related quality of life [18, 82–84]. Our results can contribute to the improvement of treatment approaches by highlighting the necessity to reduce elevated preoperative stress. Among the psychological treatments that have shown to increase SOC and meaning in life are mindfulness-based and existential therapies. It would be worthwhile to explore to which extent these interventions are feasible and effective in acute and time-restricted preoperative settings. Nevertheless, it is also important to include interventions that do not primarily aim at increasing dispositional SOC or meaning in life. These interventions would explicitly support patients with low SOC by developing a trustful situational approach that communicates a preoperative atmosphere of meaningfulness, comprehensibility, and manageability. Within patient-centered communication, individual worries, doubts, and misunderstandings concerning the upcoming surgery should be explored and clarified. This may comprise providing medical information on specific surgeries and their chances of success, but most importantly, clarifications of the individual meaning of surgical procedures, and their necessity.

To conclude, we investigated a large sample (*n* = 4918) of patients with malignant, benign, and without neoplasms. In all three patient groups, SOC was directly associated with perceived hospital and surgery related stress, with higher SOC being related to lower stress. These results can inform treatment approaches to reduce elevated preoperative stress by specifically addressing low SOC.

## Data Availability

Datasets of this study are not publicly available because study participants did not give their approval.

## Abbreviations

*ASA*: American Society of Anesthesiologists
*BASOC*: Brief Assessment of Sense of Coherence
*BRIA*: Bridging Intervention in Anesthesiology
*CCI*: Charlson Comorbidity Index
*IQR*: Interquartile range
*OLS*: Ordinary least square
*POSSUM*: Physiological and Operative Severity Score for the enUmeration of Mortality and Morbidity
*SOC*: Sense of Coherence
*SOC-29*: Sense of Coherence Scale 29
*SoMe*: Sources of Meaning and Meaning in Life Questionnaire
*STAI*: State Trait Anxiety Inventory
*WHO-5*: World Health Organization 5-item Well-Being Index
